# Rare, long-term complication after pancreatoduodenectomy—a case report of cecal volvulus

**DOI:** 10.1093/jscr/rjab202

**Published:** 2021-05-28

**Authors:** Colleen P Nofi, Caroline Maloney, Michelle P Kallis, Anna T Levy, William H Nealon, Matthew J Weiss, Danielle K DePeralta

**Affiliations:** Northwell North Shore/Long Island Jewish, Department of Surgery, Queens, NY 11040, USA; Northwell North Shore/Long Island Jewish, Department of Surgery, Queens, NY 11040, USA; Northwell North Shore/Long Island Jewish, Department of Surgery, Queens, NY 11040, USA; Northwell North Shore/Long Island Jewish, Department of Hematology/Oncology, Queens, NY 11040, USA; Zucker School of Medicine at Hofstra/Northwell, Hempstead, NY 11549, USA; Northwell North Shore/Long Island Jewish, Department of Surgery, Queens, NY 11040, USA; Zucker School of Medicine at Hofstra/Northwell, Hempstead, NY 11549, USA; Northwell North Shore/Long Island Jewish, Department of Surgery, Queens, NY 11040, USA; Zucker School of Medicine at Hofstra/Northwell, Hempstead, NY 11549, USA; Northwell North Shore/Long Island Jewish, Department of Surgery, Queens, NY 11040, USA; Zucker School of Medicine at Hofstra/Northwell, Hempstead, NY 11549, USA

## Abstract

Complications after pancreatoduodenectomy are common, and range widely in timing of presentation, relation to pancreatobiliary pathology, and necessity of operative intervention. We present a case of a 74-year-old male with history of pancreatoduodenectomy for pancreatic adenocarcinoma who presented 11 months after index operation with cecal volvulus and required emergent right hemicolectomy. Prior history of pancreatoduodenectomy with mobilization of the right colon likely predisposed him to development of this surgical emergency. Patients have altered gastrointestinal anatomy after pancreatoduodenectomy and special care is necessary to protect the afferent biliopancreatic limb during intraoperative exploration, and particularly if right colectomy is necessary.

## INTRODUCTION

Pancreatoduodenectomy (PD) is associated with high perioperative morbidity [1]. Complications after PD including pancreatic fistula, delayed gastric emptying and postpancreatectomy hemorrhage have been well-described; however, there has been less emphasis on delayed, nonhepatobiliary complications in the literature [2]. To our knowledge, large bowel obstruction (LBO) secondary to cecal volvulus has not been described as potential late complication of PD. Although rare, cecal volvulus should be considered as potential cause for obstruction, especially following surgical procedures that utilize right colonic mobilization [3]. Given the potential for catastrophic complication if diagnosis is delayed, high index of suspicion is needed for patients with relevant surgical history, such as PD. [4] Furthermore, knowledge of post-PD anatomy is critical for safe intraoperative management. We present a case report of cecal volvulus occurring 11 months after PD.

## CASE REPORT

**Figure 1:**
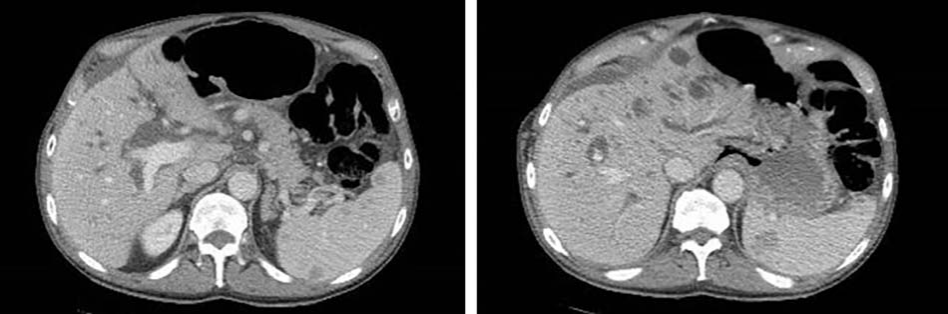
CT of the abdomen and pelvis demonstrating severe intrahepatic and extrahepatic biliary ductal dilation and multiple splenic hemangiomas.

A 74-year-old male with past medical history of hypertension, coronary artery disease and prostatectomy, presented with jaundice, weight loss and abdominal pain. Initial cross-sectional imaging revealed biliary ductal dilation and a stricture of the pancreatic portion of the common bile duct (Fig. 1). Endoscopic retrograde cholangiopancreatography was performed, at which time biopsy was nondiagnostic. After a 6-week delay, the patient underwent PD for presumed malignancy, with final pathology revealing moderately differentiated invasive pancreatic adenocarcinoma, T2N1, with 2 of 22 lymph nodes involved, and negative margins. Of note, the right colon was mobilized from its lateral attachments and the afferent biliopancreatic jejunal limb was passed through the transverse mesocolon to the right of the middle colic vessels. Postoperatively, the patient completed 6 months of adjuvant chemotherapy with FOLFIRINOX. There was no evidence of recurrence on surveillance imaging.

**Figure 2:**
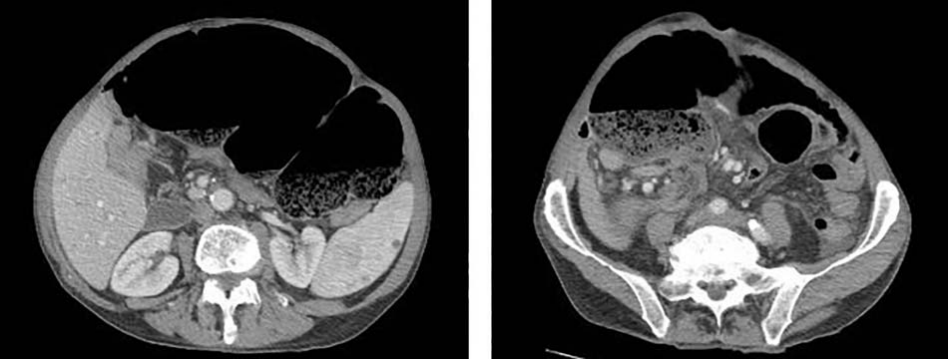
CT of the abdomen and pelvis demonstrating coffee-bean shaped cecum in left upper quadrant dilated to 12 cm, consistent with cecal volvulus.

Eleven months after PD, the patient presented to the emergency department with abdominal pain, distension and obstipation. He was hemodynamically stable, with physical examination notable for large incisional hernia with severe abdominal distension and tenderness without peritonitis. Computed tomography scan of the abdomen demonstrated a coffee-bean shaped cecum in the left upper quadrant with dilated colon and evidence of mesenteric swirling in the right lower quadrant, consistent with cecal volvulus (Fig. 2). The patient was taken to the operating room for urgent exploration.

**Figure 3:**
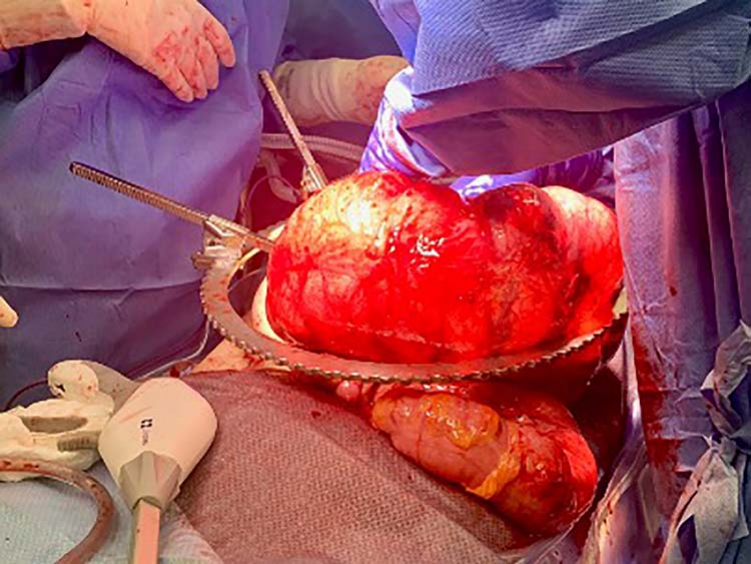
Intraoperative picture of cecal volvulus.

Exploratory laparotomy revealed a massively dilated cecum and right colon with complete 360-degree clockwise rotation along the ileocolic pedicle. The right colon and cecum were eviscerated from the abdomen (Fig. 3). The colon was viable, but extremely dilated and thin walled. The proximal region of the volvulus was identified, and the distal ileum was divided. A decompressed region of transverse colon was identified as the distal region of the volvulus. There were extensive adhesions involving the afferent biliopancreatic limb and the gastrojejunal anastomosis to the transverse mesocolon. Once mobilized, the transverse colon was divided to the right of the middle colic vessels. The specimen was detorsed immediately prior to division of the ileocolic pedicle, and ileocolic anastomosis was performed. Four small (<2 cm) uninvolved peritoneal implants were identified, removed and sent for permanent pathology.

The patient tolerated the operation well, his postoperative course was uncomplicated, and he was discharged on postoperative Day 3. Pathology from the peritoneal implants revealed moderately differentiated adenocarcinoma compatible with metastatic pancreatic cancer. The terminal ileum, cecum, appendix and ascending colon were negative for malignancy.

## DISCUSSION

Cecal volvulus is an uncommon cause of intestinal obstruction [5, 6]. Previous abdominal surgery has been identified as a risk factor for cecal volvulus [7]. It is intuitive that operations that mobilize the right colon by taking down the lateral retroperitoneal attachments could predispose patients to cecal volvulus by increasing the mobility of the right colon [3]. Mobilization of the right colon is frequently performed through Cattell–Braasch maneuver during PD, commonly done in patients with borderline resectable or locally advanced tumors to improve exposure [8]. In addition to anatomic considerations, many patients with cecal volvulus report history of chronic constipation or gastrointestinal dysmotility [4].

Post-PD anatomy is complex and understanding of altered anatomy is crucial when planning operative reintervention. In PD reconstruction, the afferent limb may be brought through either the ligament of Trietz defect or transverse mesocolon. Understanding the location of the afferent biliopancreatic limb is of particular importance in subsequent operations, as it will be encountered when mobilizing and dissecting free the transverse colon, as is necessary in performing a right hemicolectomy. Postoperative adhesions involving the afferent limb and biliopancreatic anastomoses may complicate dissection, and careful lysis of adhesions is imperative in order to protect these structures. Whenever possible, an experienced pancreatic surgeon should be involved in patients requiring reoperation after PD.

Much of the literature evaluating the morbidity associated with PD focuses on hepatobiliary complications, including delayed gastric emptying, pancreatic fistula, pancreatic leak, and gastroduodenal artery pseudoaneurysm and hemorrhage. Few studies have focused on nonhepatobiliary, delayed complications. Late complications (those occurring >90 days after index operation) may occur in as high as one-third of patients undergoing PD, which include incisional hernia, biliary stricture, cholangitis, pancreatitis, small bowel obstruction and peptic ulcer [2]. Of all patients undergoing PD, nearly one-fifth may late reinterventions with percutaneous, endoscopic or surgical procedures [2]. In contrast to early complications, late complications tend to require more invasive interventions [2, 9, 10]. Large bowel volvulus has not yet been described as potential long-term complication.

In patients that have previously undergone PD for cancer, late complications often occur in the setting of recurrence, and surgical exploration may lead to the diagnosis of radiographically occult recurrent malignancy [11, 12]. In these cases, it is important to employ a multidisciplinary treatment approach that balances disease control, quality of life and patient preference [13]. The decision whether to pursue additional systemic therapy for asymptomatic and radiographically occult recurrence can be challenging. In this case, gemcitabine and nab-paclitaxel was initiated with the goal of delaying disease progression and prolonging survival. Unfortunately, the patient developed rapidly progressive carcinomatosis and enrolled in hospice 4 months after cecal volvulus.

The high incidence of complications after PD and rates of recurrence for malignancies treated with PD highlight the importance of frequent and long-term follow-up after index operation. Specifically, cecal volvulus may present as a long-term sequela after PD and should be included in the differential for obstruction in the post-PD patient.
